# “No One Can Tell Me How Parkinson’s Disease Will Unfold”: A Mixed Methods Case Study on Palliative Care for People with Parkinson’s Disease and Their Family Caregivers

**DOI:** 10.3233/JPD-212742

**Published:** 2022-01-21

**Authors:** Herma Lennaerts-Kats, Anne Ebenau, Jenny T. van der Steen, Marten Munneke, Bastiaan R. Bloem, Kris C.P. Vissers, Marjan J. Meinders, Marieke M. Groot

**Affiliations:** a Department of Neurology, Radboud University Medical Center, Donders Institute for Brain, Cognition and Behaviour, Nijmegen, The Netherlands; b Department of Anesthesiology, Radboud University Medical Center, Pain and Palliative Care, Nijmegen, The Netherlands; c Department of Primary and Community Care, Radboud University Medical Center, Nijmegen, The Netherlands; d Department of Public Health and Primary Care, Leiden University Medical Center, Leiden, The Netherlands; e Radboud University Medical Center, Radboud Institute for Health Sciences, Scientific Center for Quality of Healthcare, Nijmegen, The Netherlands

**Keywords:** Parkinson’s disease, case study, palliative care, family caregivers, patients, mixed methods

## Abstract

**Background:**

Palliative care for persons with Parkinson’s disease (PD) is developing. However, little is known about the experiences of patients with PD in the palliative phase and of their family caregivers.

**Objective:**

To explore needs of patients with PD in the palliative phase and of their family caregivers.

**Methods:**

A mixed methods case study design. Health care professionals included patients for whom the answer on the question “Would you be surprised if this patient died in the next 12 months?” was negative. At baseline, and after six and twelve months, we conducted semi-structured interviews with patients and caregivers. Participants completed questionnaires on quality of life, disease burden, caregiver burden, grief, and positive aspects of caregiving. We analyzed quantitative data using descriptive statistics, while we used thematic analysis for qualitative data.

**Results:**

Ten patients and eight family caregivers participated, of whom five patients died during the study period. While the quantitative data reflected a moderate disease burden, the qualitative findings indicated a higher disease burden. Longitudinal results showed small differences and changes in time. Patients reported a diverse range of symptoms, such as fatigue, immobility, cognitive changes, and hallucinations, which had a tremendous impact on their lives. Nevertheless, they rated their overall quality of life as moderate to positive. Family caregivers gradually learned to cope with difficult situations such delirium, fluctuations in functioning and hallucinations. They had great expertise in caring for the person with PD but did not automatically share this with health care professionals. Patients sensed a lack of time to discuss their complex needs with clinicians. Furthermore, palliative care was rarely discussed, and none of these patients had been referred to specialist palliative care services.

**Conclusion:**

Patients with PD experienced many difficulties in daily living. Patients seems to adapt to living with PD as they rated their quality of life as moderate to positive. Family caregivers became experts in the care for their loved one, but often learned on their own. An early implementation of the palliative care approach can be beneficial in addressing the needs of patients with PD and their family caregivers.

## INTRODUCTION

The prevalence is rising of people with Parkinson’s disease (PD) is rising [1, 2]. Despite continued medical advances, curative treatment for PD is not yet available. Since 2004, palliative care for people with PD and family caregivers is widely advocated [3–5]. Nevertheless, it is less commonly provided for people with PD than, for example, people with a malignant disease [6], possibly because PD is not considered a ‘malignant’ disease associated with early death. Comparisons between patients with end-stage cancer and patients with PD indicate similar levels of disability and decrease of quality of life [7, 8]. Furthermore, studies comparing PD with other non-cancer diseases, such as chronic respiratory illnesses, showed that patients with PD received fewer drugs for palliation [9, 10].

Early qualitative studies have shown that patients with advanced PD have palliative care needs in light of high symptom burden and emotional distress, among other things, as well as unmet information and communication needs [11–15]. Besides, there is an urgency to speak about wishes and preferences including end of life topics earlier in the disease trajectory progressive cognitive impairment or communication problems develop [16, 17]. Although cognitive impairment may be present from the time of diagnosis, many patients develop a more severe cognitive impairment and dementia later on [5, 18, 19]. As a result, health care related issues and end of life decision-making at the end stage of PD is often left to family caregivers, who are coping with their own emotions, and often feel overburdened [11, 13, 15, 20]. Studies in other fields have shown that timely identification of patients’ palliative care needs improves satisfaction with health care, decreases unnecessary acute interventions and improves quality of life. Further, if patients’ needs are identified timely, they more often die at their preferred place [21, 22].

Inherent to the nature of PD, palliative care provision might be needed from the time of diagnosis. Research of patients’ and family caregivers’ needs in the palliative phase is scarce. The available relevant studies are limited in that they either used a retrospective design or conducted interviews at only one time point. For this study, we defined a palliative phase as a period during a PD trajectory in which care goals mainly focus on comfort [16]. This study aims to identify experiences and needs of patients with PD in the palliative phase and of their family caregivers over a 12-month period.

## METHODS

### Setting

We used a mixed methods case study design to explore the palliative care needs of patients with PD and their family caregivers, who were followed for twelve months. Their experiences throughout the palliative phase [16] were described along with factors influencing these experiences [23–26]. Combining quantitative data with qualitative data allowed us to gain a deeper understanding of experiences in the palliative phase. Data were collected between March 2018 and December 2019.

### Participants

We aimed to include five to fifteen patients with PD and their family caregivers from hospitals, general practices, and nursing homes. Potential participants were identified by healthcare professionals, including nurse practitioners, PD nurse specialists from outpatient clinics and home care organizations, nurse practitioners and neurologists, who screened their patient populations for anyone meeting the following inclusion criteria: 1) 18 years and older; 2) cognitively able to complete questionnaires and interviews; 3) diagnosed with ’idiopathic PD’ according to the diagnostic criteria of the UK Parkinson’s Disease Society Brain Bank; and 4) assumed to be in the palliative phase based on a negative answer on the question “Would you be surprised if this patient died in the next 12 months?” [27]. Family caregivers were eligible if they were: 1) 18 years and older; 2) cognitively able to complete questionnaires and interviews; and 3) identified by the patient as their family caregiver or other informal caregiver; volunteers excluded. A purposive sampling strategy was employed to include a diverse range of patients regarding gender, place of living, eligibility, and demographic characteristics. Potential study participants were contacted by their own healthcare professionals. Fourteen patients were screened by the project team to recruit a purposive sample of 10 patients. Two did not met the inclusion criteria and the other two were not included due for organizational reasons. If a recruited patient with PD died within the study’s timespan, the family caregivers were asked for informed consent to an after-death interview [28].

Ethics approval for this study was received from the ethics committee Arnhem-Nijmegen [number; 2016–2424]. Patients with PD and family caregivers received oral and written information about the study and their informed consent was obtained. Participants were not compensated for their contributions to this study. All personal data, such as, names were coded. Written informed consent forms and questionnaires were stored in a safe and locked closet and electronic data (interview transcripts) were stored in password-protected documents on a safe Radboudumc server.

### Data collection

#### Qualitative data

Data were collected by semi-structured face-to-face interviews by HL or MG—both researchers trained and experienced in qualitative research—at baseline (T0), after six (T1), and twelve months (T2). At T1 a telephone interview was held with family caregivers only to enable them to speak freely without the presence of the person with PD. The topic guide consisted of the themes and subthemes described in Table 1. The topics were derived from a literature study, as well as from the expertise of the members of the project group, including senior researchers, a PD nurse specialist, physicians, and a member of the Dutch Association of Parkinson’s Disease in the role of family caregiver. The latter reviewed the interview topics and adjustments were made according to the suggestions she provided. During the interviews, patients with PD were often joined and supported by the family caregiver to help clarifying communication, if necessary. The patient was first interviewed (duration: 20–35 min), and thereafter the family caregiver (duration: 30–75 min). Interviews were held at the patient’s residence, either the private home or nursing home. After-death interviews with caregivers were conducted by telephone (duration 45–60 min). There was no prior relationship between the interviewers and the participants; the interviewer stated her name and occupation before the interview started.

**Table 1:** Topics of individual interviews and after-death interview

(sub)Topics for individual interviews	Patient	FC
1: Experiences with advanced PD and palliative care
Patients’ symptoms and needs on the physical, psychosocial and spiritual domain	x	x
Family caregiver’s needs on the physical, psychosocial and spiritual domain		x
Care for a person with PD: communication, changes in a person health status, demands on family caregivers		x
Conversations and information about prognosis, medical options, end-of-life care and treatment by healthcare professionals	x	x
Changes in care: transition to palliative phase, care goals	x	x
Future preferences, expectations and hope	x	x
2: Documentation of personal wishes
Declarations regarding treatment (cure), care, dying, funeral, representative	x	x
(in)formal support	x	x
3: Professional support
Involvement in care and decisions	x	x
Healthcare professionals’ expertise, availability, information provision; care coordination	x	x
Healthcare professionals’ attention and commitment to patient as a person and social context	x	x
Professional support for family caregiver		x
(sub)Topics for after-death interviews
Identification of the palliative phase: explication of and communication about the palliative phase and possibilities by healthcare professionals		x
Primary care process: care for multidisciplinary needs, contact with healthcare professionals, expertise and knowledge of healthcare professionals		x
Communication and involvement of family caregivers in care and decisions		x
Communication with patient		x
Patient’s autonomy: following his or her preferences		x
Dying and funeral		x
Care for bereaved family caregivers: aftercare		x

#### Quantitative data

Data were collected at baseline, T1, and T2 using demographics and questionnaires; Hoehn & Yahr scale [29], Schwab & England scale [30], and Unified Parkinson’s disease rating scale (UPDRS) part 1 [31]. These self-report questionnaires were administered after the interview with the patient, or—if he or she was too tired—at a later stage. The following questionnaires were used for patients with PD: Parkinson’s Disease Questionnaire (PDQ-8) [32], Edmonton Symptom Assessment Scale - Parkinson Disease (ESAS-PD) [8], and Functional Assessment of Cancer Therapy–General (FACT-G) [33]. Family caregivers completed a Marwit-Meuser Caregiver Grief Inventory (MM-CGI-SF) [34], Zarit Burden Interview (ZBI) [35] at baseline, T1, and T2, and the Positive Experiences Scale (PES) at T1 and T2. Details on the instruments are described in Supplementary Material 1.

### Data analysis

For the data analysis, we used a convergent design with the qualitative and quantitative dataset. The two data sets were first analyzed separately. Thereafter, results were merged for comparison by a ‘weaving approach’, which involves combining qualitative and quantitative data [36]. Quantitative data are described under the respective themes identified from the analysis of the qualitative data.

#### Quantitative data

Quantitative data are summarized using mean or median scores and underlie the descriptions of the characteristics and changes of the cases in time.

#### Qualitative data

Data was recorded and transcribed verbatim by transcription services. We used at thematic content analysis for identifying themes across the data set [24–26]. Driven by the data, seven interviews (with four patients and three caregivers) were coded independently by HL and AE (second author, trained and experienced in qualitative research). They compared and fine-tuned their codes on an abstraction level till intercoder agreement was achieved. Third, all *in-vivo* codes were compared, connected, and finally clustered into themes and subthemes by HL and AE. Afterwards, themes and subthemes were noted in a codebook, which was ‘checked’ in relation to the remaining interviews. It served as the coding strategy, which was further adapted if new subthemes or codes came up during data analysis. HL and AE coded two new interviews with the codebook and checked for intercoder agreement, which again was high. Next, they analyzed the remaining interviews. This procedure was followed first for the baseline interviews. Thereafter, T1 and T2 interviews, too, were analyzed with this codebook to check for congruence and difference between themes/subthemes and change over time. If data did not fit the initial coding scheme, new themes and or subthemes were formulated and added. Ultimately, analysis of fourteen interviews resulted in a final, clear-cut codebook. Data saturation was reached when no new subthemes came up for each time moment (baseline, T1, and T2). The coding process was supported by the qualitative data analysis & research software Atlas.ti 8.4.

## RESULTS

Ten patients with PD and eight family caregivers were included based on the inclusion and exclusion criteria, were willing to participate and gave informed consent. In six cases, a patient and family caregiver participated; in three cases, the patient only; and in one case, a patient and two family caregivers. In total, 43 questionnaires were filled in and 33 interviews were administered (thirteen baseline interviews, ten T1-interviews, seven T2-interviews, and three after-death interviews). The questionnaire completion rate was 100%for both patients and family caregivers.

The median age of the patients was 77 years, that of the family caregivers 75 years (Table 2). Five patients died during the study period: two from pneumonia (*n* = 2), one from heart failure, one from a neck fracture and one of organ failure. Two family caregivers were lost from the study after the family member with PD died. One of them refused consent for an after-death interview; the other died within a month after the death of the family member with PD.

**Table 2:** Participants characteristics at baseline

	Patients with PD	Family caregivers
Number of participants, n	10	8
Women, n (%)	6 (60)	1 (12)
Age, median (range)	77 (68–82)	75 (52–84)
Disease duration years, median (range)	11 (5–29)	NA
Relationship, n (%)
Spouse	–	7 (88)
Son	–	1 (12)
Highest level of education, n (%)
Secondary education	6 (60)	1 (12)
Higher education	4 (40)	6 (76)
University	–	1 (12)
Presence of deep brain stimulation, n (%)	1 (10)	NA
Living situation, n (%)
Nursing home	4 (40)	–
Home	6 (60)	10 (100)
Involved healthcare professionals, n (%)		NA
Physiotherapist	9 (90%)
Neurologist	8 (80%)
PD nurse specialist	8 (80%)
Community nurse	8 (80%)
General practitioner	6 (60%)
Elderly care physician	4 (40%)
Speech therapist	2 (20%)
Occupational therapist	2 (20%)
Dietician	1 (10%)
Psychiatrist	1 (10%)

Two patients died at home and three in the nursing home in which they already lived when they entered the study. The period from inclusion until death ranged from 2 to 11 months with a median of 8 months. Before death, there were no hospital admissions in these five cases. None of the patients had been referred to specialist palliative care services.

Below, the four main themes that resulted from data analysis are described. Both quantitative and qualitative results showed small differences and changes over time, which we considered negligible.

### Theme 1: Burden of PD

Eight patients were rated as Hoehn & Yahr stages 4 or 5 (Table 3). They were severely disabled or even wheelchair bound. All reported a variety of symptoms but rated their symptom burden low on the ESAS-PD. Fatigue, sleep difficulties, communication issues, falls and freezing were the symptoms described as common and distressing aspects of living with advanced PD.

**Table 3:** Results of questionnaires

	Baseline	T1	T2
Patients, n	10	7	5
Hoehn & Yahr, n (%)
Possible range: 0–5 (higher scores: higher stage of disease)			
3	2 (20)	1 (14)	1 (20)
4	5 (50)	4 (57)	3 (60)
5	3 (30)	2 (29)	1 (20)
Schwab & England, mean,±SD;
Possible range: 0–100 (higher scores: higher levels of dependency)			
	44.0±17.1	38.8±18.9	42.0±19.2
UPDRS, median;
Possible range: 0–4 (higher scores: higher levels of motoric and behavioral problems)			
Intellectual impairment	1	2	2
Thought disorder	1	1	2
Depression	1	2	1
Motivation	1	1	2
PDQ-8, mean,±SD
Possible range: 0–100 (higher scores: lower levels of overall health)			
	33.5±12.3	35.7±10.0	37.5±6.1
ESAS-PD, mean,±SD
Possible range: 0–140 (higher scores: higher symptom burden)			
	38.3±11.8	39.0±13.8	45.4±11.0
FACT-G, mean,±SD
Possible range: total 0–108, physical well-being 0–28, social/ familial well-being 0–28, emotional well-being 0–24 and functional well-being 0–28 (higher scores: worse physical and emotional well-being and better social and functional well-being)			
Total score	75.6±5.2	69.6±6.7	72.3±7.7
Physical	16.1±2.0	13.7±2.6	16.5±3.1
Social/familial	23.4±3.3	19.1±4.3	20.5±5.8
Emotional	15±2.8	14.1±3.1	15.8±3.3
Functional	21.1±3.9	22.7±3.3	19.5±2.5
Family Caregivers, n	8	7	5
ZBI, mean,±SD
Possible range: 0–88 (higher scores: higher levels of self-rated burden)			
	30±10.3	26.3±12.1	31.8±11.1
PES, mean,±SD
Possible range: 0–24 (higher scores: higher level of positive experience)			
	–	20.9±3.2	19.6±4.0
MM-CGI-SF, mean,±SD
Possible range: 0–90 (higher scores: more self-rated pre-death grief)			
	49±12.4	49.2±12.1	43±11.9

ESAS-PD, Edmonton Symptom Assessment Scale Parkinson Disease; PDQ, Parkinson’s Disease Questionnaire; FACT-G, Functional Assessment of Cancer Therapy –General; MM-CGI-SF, Marwit-Meuser Caregiver Grief Inventory Short-Form; PES, Positive Experiences Scale; UPDRS, Unified Parkinson Disease Rating Scale; ZBI, Zarit Burden Interview.

*Look, at this point I have commonly freezing moments. In my mind, I am already walking, but my feet are still at the same place. At one point, I fell through the window (patient)*

Symptoms like these make it difficult for patients to maintain their daily activities. In particular, their ability to communicate worsened, which had a great impact on social roles, relationships, and connectedness with the outside world.

*I have been among people all my life, and now you feel excluded*. . .*. that you can’t have a conversation with people anymore. (patient)*

Patients needed to rely on others for activities such as visiting friends or shopping. Adhering to medication schedules, in some cases 8 to 10 medication intakes a day, was perceived as a challenge. Some patients also sensed that others did not like to be confronted with advanced PD, which led to social isolation.

*Other people think that nothing is wrong. First of all, when she talks with difficulty, the first reaction is, hey? What are you saying? Can’t you speak any clearer? And if you then fall asleep, they’re like, I’m never coming here again. (caregiver)*

Patients and their family caregivers had to adapt continuously to a new health status and accept losses in functioning. Fluctuations in functioning and health occurred from hour to hour in some cases, which was difficult to cope with. Several emotions were mentioned, such as frustration, sadness, and fear of the future. Some patients somehow “lost themselves” and did not recognize themselves in their current situation.

*If I want to see the children, all that has to be organised in a special way.*

### So how does that affect you?

*Well, I find that very annoying... I used to be so active, it feels as if it doesn’t reflect on me at all. (patient)*

Still, patients described that, to some extent, they had gotten used to their current situation, and could enjoy “little things”, such as a visit from family, music, or a short walk. The mean FACT-G total score implies that patients, generally, perceived their quality of life as quite moderate or positive instead. Overall, health-related quality of life on the PDQ-8 was low.

### Theme 2: Professional healthcare

Patients indicated difficulties obtaining access to various medical services. More specifically, they mentioned the waiting lists and the scheduled times of appointment with their neurologist; patients experienced off periods during the day which makes it difficult to keep an appointment.

*I would like to have the opportunity tomorrow, if only for 10 minutes, for [neurologist] to see how I am now. You just don’t get in...If I call the hospital, I want to see Dr [neurologist], it’s the next month. There is never an instant possibility, You have to so see how I feel now and what is going on. Then you can come in there in 2 or 3 weeks, and then the situation is totally different. And then they say, what are you here for? (patient).*

Sufficient time during doctor-patient conversations was an unmet need, especially when end-of-life issues were addressed. Patients desired to be listened to by healthcare professionals about their individual, fluctuating needs. Understanding the person behind the patient was felt essential for delivering person-centered care. Furthermore, they wished for honest and open conversations on their current and future health situation. However, sharing experiences was often hindered by patients’ communication problems.

*But you come into a doctor’s office, they then first take minutes to remember who is that again. There is hardly any recognition or personal recognition. There is only recognition through that computer with all those numbers on it. Especially because in 10 minutes, you sit, on the edge of the chair to say exactly what I say, because otherwise I have too little time to say what I want. You’re already going into a kind of, constriction by tension. 10 minutes is nothing at all*! *Especially with a Parkinson’s patient, because there’s nothing she can do about it. But she often can’t find the right words to say what exactly she means. The minutes then go by... Yes, I take over to explain what she means. You never get an honest conversation. (caregiver)*

Many participants had perceived a lack of continuity or coordination of care, both within and between health care services. Lastly, the frequent medical appointments associated with PD or co-morbidities necessitate transport to reach the hospital. Patients, therefore, were dependent on their relatives or public transportation, which was sometimes troublesome and a reason to cancel an appointment. Also, patients and family caregivers believed that there were fewer medical options left to alleviate the current symptoms.

*You can decrease the medication and you can increase the medication, but I think that there is not much else you can do, at this stage. You can still change something in the settings, but that... (caregiver)*

However, instead of grief, most participants seemed to feel resigned as a result of living for so long with PD. Some patients stopped visiting an outpatient clinic as a consequence.

### Theme 3: Support by family caregivers

Family caregivers reported numerous everyday tasks, such as feeding, dressing, and helping their loved one to bed.

*I have to lift her out of the bed to the toilet. [*. . . *] Sometimes she made it, sometimes she didn’t make it. Or she was in the toilet, and then she didn’t have to go anymore and then back again. Putting her back to bed. How often did this happen per night? Sometimes up to 12 times... (caregiver).*

They also have a role in arranging, coordination and organization of healthcare, without little support from health care professionals. Family caregivers said that they had not been informed about what was available for them in forms of information or professional healthcare, but that, instead, they had found out by chance.

*We were never told that with Parkinson’s you also suffer other effects over time. ***And you mean those cognitive changes***. Yes and you don’t know that. [*. . . *]Now the penny drops again when the doctor says: ‘Madam is like this and like that’. Pennies have regularly dropped in cases when you only hear something afterwards, a remark that makes you say: ‘oh I know this’. [. . . ]*

Family caregivers reported a continuous state of awareness as a burden. The continuous awareness was needed to guarantee the patients’ security and prevent crisis situations.

*Yes, you have no rest. Then you think what is she going to do, she’s not going to get up, is she? And constant restlessness that she might fall or something, you know. (caregiver)*

Family caregivers had been taking care of their loved one for a long period, and consequently had developed a way of caregiving tailored to the loved on. They had learned how to deal with difficult situations such as delirium, hallucinations and had found ways that helped them cope with difficult situations. Their expertise was acquired from previous experiences, and they had developed creative solutions for everyday problems; for example, do not argue but confirm beliefs. Although these family caregivers acquired much knowledge and expertise about the person with PD, they did not mention sharing this with healthcare professionals.

Family caregivers described their care giving as “a duty”. Some of them were almost overwhelmed by the promise they had made to take care of their loved one.

*I think I am obliged to. You married her, she gave you children, you can’t just abandon her, that’s how I feel. (caregiver)*

In general, though, scores on the ZBI indicate that family caregivers experienced mild to moderate burden during the entire time span of participation. A few family caregivers talked about their feelings of being overburdened the period before the loved one had been placed in a nursing home. Their current caregivers’ burden was rated lower than that of family caregivers who were living with the patient at home, probably because they no longer played a role in daily care. Scores on the MM-CGI-SF show that pre-death grief is average among family caregivers of patients with PD. Furthermore, results from the PES indicate a high level of positive aspects of family caregiving (Table 3).

The three bereaved family caregivers felt that current life was a balancing act between ‘mourning’ and ‘continuing’, which was sometimes a lonely journey. Two of them mentioned that death was a relief for the patient –and similarly also for them.

*She did not want to become like other people in the nursing home. For the rest, she may have been saved from a lot of suffering.... I am glad that she did not suffer. (caregiver)*

The funeral was described as a “beautiful, satisfying and warm memory” of paying the last respect to the person. Important contributors to that feeling were raising memories of the person with closest family and friends, in concordance with the person’s lifestyle and wishes, and at a place that was familiar for the person, i.e., the nursing home. Shortly after the death of the loved one, participants received informal support from their family and friends, but this slowly petered out. Bereaved family caregivers had not received oral or written information about grief or bereavement support.

### Theme 4: Thinking about the future

Some participants had given little thought to advance directives or end-of-life decisions. Other responded to the question on thinking about the future as “we cannot predict” and with a ”living by the day” attitude.

****But has it been discussed what is going to happen in the future?*** Oh, they don’t know that either... Everything can happen to you that doesn’t happen to others. Then you are an exception to the rule maybe. ***How do you look at it?*** Yes, we will see. You cannot predict this. You should not worry about it. It passes in your mind, but it doesn’t bother me. (patient)*

*No one can tell me how PD unfolds; that is still uncertain*. . . *(patient)*

Some mentioned a readiness to make these decisions but had not yet acted upon it. A few participants had shared specific needs and wishes with their family or general practitioner. Patients and family caregivers wished for timely discussions about prognosis, future symptoms, medical treatment, and reimbursement. We discerned that healthcare professionals, most often neurologists, rather casually informed the patients about medical options for advanced PD. Furthermore, options of palliative and supportive care were rarely discussed. Patients appeared more likely to discuss end-of-life care in times when illness worsening restricted their way of life. If these discussions took place, this not automatically meant that a living will or advance directive had been prepared. Conversations about future care happened in most cases ‘with death in mind’. A few participants did speak about end-of-life issues in case they should have reached the terminal phase, but not about the period between now and the terminal phase.

Participants made clear that when medical treatment had become futile, they did not want to be distressed by life-prolonging interventions such as resuscitation, tube feeding and hospitalization. Some said they would wish to hasten death by means of euthanasia when they became more ill.

*I asked her when euthanasia? Yes, we talked about that and her wish to get euthanasia if things more worsened. Well, that gives me a peace of mind, because she is going to a direction were several symptoms will occur. (caregiver)*

Often, participants’ ideas about end-of-life issues were based on experiences of others, without having information on or having discussed this topic with health care professionals.

*The woman who has always been neat and tidy, she can’t live alone in [place name] anymore. She has been placed in the home as the only woman without dementia on a ward. No lock on the door, well I, she said I would rather have died than be here. (patient)*

Substantively, this focused on treatment or state of life that was wished to be avoided.

## DISCUSSION

This study highlights the experiences of patients with PD in the palliative phase and their family caregivers over a 12-month period. The qualitative and quantitative findings were mixed and interpreted together. We found that a variety of symptoms had an impact on patients’ and family caregivers’ daily functioning in all life domains—physical, psychological, existential, and social.

Patients were dependent on others to maintain daily activities and had faced severe losses in the preceding years. Often, they and their family caregivers had learned to accept this, or at least had resigned themselves to it. Patients enjoyed ‘little things’ in life, such as listening to music or being together with family. The qualitative part of the study showed several burdensome symptoms, yet the quantitative data showed that the symptom burden was not assessed as extremely high. We assume that patients might underscore their symptom burden when they have resigned themselves to the condition or lack self-awareness, like reported in other studies of patients with PD [37–39]. Cognitive deficits, apathy or mood disorders might negatively affect self-awareness, too. We have not sufficient data, however, to confirm this supposition. The findings of this study are consistent with those of previous studies in PD, in that multiple symptoms affect the daily life of a person with advanced PD [40–42]. However, these studies often found a high symptom burden and low quality of life, which we did not find in our group of participants. A possible explanation might be a response shift and the fact that physical symptoms are not the only factors influencing patients’ quality of life [40, 43, 44]. Spiritual and psychological care are equally important for them and their caregivers [40, 45].

Findings of our study emphasize the need for improving palliative care for people with PD. In particular, participants noted that specialists reserve little time for consultations, while they have complex needs. Furthermore, long waiting lists for consultations were seen as a barrier, especially when one’s health status deteriorated. Besides, travel distance and difficulty with public transportation were mentioned as reasons for cancelling appointments. We suggest that telemedicine or telehealth can improve specialist care for patients with PD. Telemedicine has already been shown to increase access to palliative care services [46, 47]. PD nurse specialists and social workers, too, play an important role in improving quality of care, by providing information, fulfilling a surveillance role, and facilitating family participation and easy accessibility to healthcare services [48–50]. However, there comes a moment when palliative care from a general practitioner is more appropriate [51]. This particular moment seemed to have gone unnoticed by the healthcare professionals in our cases, whereas patients and family caregivers felt that this moment had already passed. Improved collaboration between general practitioners and neurologists might be helpful, as both types of clinicians play a pivotal role in caring for a person with PD, until death [51]. Additionally, other models might be considered, including consultative palliative care teams, integrated palliative care programs and complementary models (including primary palliative care, an acute palliative care unit, and an outpatient supportive care clinic) [4, 5, 52, 53]. A promising recent randomized clinical trial on the integration of outpatient palliative care showed positive outcomes on quality of life and better symptom burden for people with PD [54]. Quality of life was improved for those in the intervention group compared to those in the control group, as well as non-motor symptom burden, motor symptom severity, completion of advance directives, caregiver anxiety, and caregiver burden at 12 months. Lastly, health care professionals should be offered more training in palliative care needs and the identification thereof, which has also been suggested in other studies in the field of palliative care for PD [4, 7, 11, 13–15, 40, 55, 56].

Family caregivers seem to regard the increasing burden as a consequence of the natural process of PD. Many caregivers provided extensive care for their relatives. Often, they overcame great challenges without receiving much formal or informal support. The family caregivers in our study did not have time and space to reflect upon the impact of caregiving and the needs they might have themselves. Besides, the caregiver burden was found to be moderate, while we had expected this to be high, considering their daily tasks. One possible explanation is that some of the participants had experienced a high burden in the period before the relative was placed in a nursing home. Another explanation is that the family caregivers’ perception of their burden had changed. Comparing one’s own health situation to that of the patient might lead to underestimating one’s situation. Furthermore, as caregivers valued the rewarding aspect of caregiving high on the PES, it might be interesting to find out whether this his rewarding aspect has a protective effect on overall burden. Our findings are in line with those of other studies that show negative consequences, such as caregivers’ isolation, burden, and health problems [7, 12–15]. The finding that caregiving is not only a burden but can also be rewarding was mainly a quantitative finding. Other studies on caring for a person with chronic disease found such benefits as well [57–59].

The family caregivers had acquired much expertise on how to take care for their relative with advanced PD. None of the participants, however, reported that they had shared this expertise with healthcare professionals, nor that they had been asked to share it. We therefore suggest that a lack of time and attention from healthcare professionals as well as little relational continuity might be reasons for not sharing important experiences. Many studies have shown the importance of supporting and involving the informal caregiver in professional healthcare for a person with a chronic disease, which support improves patients’ and family caregivers’ satisfaction [60–62].

Bereavement support consisted of an expression of sympathy from the health care professional involved, who, however, did not provide information about grief, bereavement, or other support services. This finding is in line with our previous qualitative interview study on the experiences of bereaved family caregivers, to the effect that bereavement programs were not routinely offered [20]. Furthermore, society cultural norms ‘to continue’ and ‘to go on’ might not be helpful in the long term.

The patients and family caregivers in the present study identified the need for timely support and information on planning for the future. However, only a minority of the patients had actually discussed their end-of-life care preferences with their relatives or GP. Furthermore, while patients and family caregivers did talk about end-of-life issues and death, they did not discuss the period between ‘now’ and ‘death’. It is probably easier to speak about end-of-life and death than about the period when a patient has become dependent on others. Alternatively, cognitive impairment might have hindered patients in foreseeing developments or in abstract thinking about the future. Advance care planning has received increased attention, and is defined as a process that enables individuals to define goals and preferences for future medical treatment and care, to discuss these goals and preferences with family and health-care providers, and to record and review these preferences if appropriate [63]. Research undertaken in other populations suggested that ACP can improve quality of care at the end of life and improve communication between patients, their families and health care professionals [64, 65]. This is particularly true for patients with cognitive impairment, who may have difficulty in communication their person’s goals or preferences [66]. Furthermore, ACP can be helpful in developing a more patient-centered care plan based on the personal goals of a patient and family caregiver [67]. By now, ACP has been recognized as helpful for people with PD to declare their wishes on future care with regard to potentially occurring situations [56, 67–71], although its effectiveness has not been shown in randomized clinical trials.

### Strengths and limitations

A particular strength of this study is that the interviews and questionnaires were administered at three points in time. The severity of the illness often precludes obtaining all desired information from a patient. Generally, the patients suffered from severe fatigue, communication problems, and a lack of concentration. The fact that we interviewed them three times helped to gain a better in-depth view of living with advanced PD. Furthermore, content validity improved by this approach as we could confirm earlier findings from interviews.

A limitation of this study is that we included a small sample of patients with PD who were cognitively capable of filling in questionnaires and answering questions from the interviewer. The generalizability of findings is also limited by the convenience nature of the sample. Furthermore, the study findings derive from patients of two different settings. We acknowledge that the context of care can make a difference, in particular with regard to the theme of professional healthcare. Also, the use of the FACT-G should be considered, as this instrument has been widely validated in cancer patients, but not in patients with PD.

Besides, the interviewers might have unintentionally focused on burden of care, which could explain the discrepancies between the qualitative and the quantitative results. Literature often describes this tendency of focusing on the negative side of caregiving [72]. Lastly, our work on bereavement was limited to only three single after-death interviews. These interviews with bereaved caregivers were held relatively soon after the relative had died, which means that we have no insights on the long-term experiences.

## CONCLUSION

This mixed-methods case study explored the experiences of patients with PD in a palliative phase and their family caregivers, over a 12-month period. Patients described many difficulties in daily living but rated their quality of life as moderate to positive. Furthermore, family caregivers were not well equipped to provide care at an advanced stage of the disease, and often learned person-centered approaches from doing in practice. They did not, however, share the gained expertise with healthcare professionals. Lastly, patients and family caregivers felt that they were in a phase in which medical options were limited. They barely addressed this with the healthcare professionals, while this could be an important trigger to open up discussions about palliative care needs.

### Future directions

This study indicates that patients’ and families’ needs are not fully addressed. Patients often needed more time for consultation, better information about and support in maintaining their daily activities. Advance care planning could benefit patients with PD, but has not been implemented in the Netherlands [17]. Ideally, ACP is started early in the disease process and at least before a patient loses decision making capacity [16]. Further research is needed on the integration of diverse models of palliative care for patients with PD and their family caregivers [54].

## Supplementary Material

Supplementary Material
