# Supplementary material for: “No One Can Tell Me How Parkinson’s Disease Will Unfold”: A Mixed Methods Case Study on Palliative Care for People with Parkinson’s Disease and Their Family Caregivers
Source: J Parkinsons Dis. 2022 Jan 21;12(1):207–19. doi: 10.3233/JPD-212742 (PMC8842750; doi:10.3233/JPD-212742)
Supplement: Supplementary Material [file jpd-12-jpd212742-s001.pdf]

Supplementary Material

"No One Can Tell Me How Parkinson's Disease Will Unfold": A Mixed Methods Case Study on Palliative Care for People with Parkinson's Disease and Their Family Caregivers

Supplementary Material 1: Instruments

1) Questionnaires for patients with PD

Parkinson's Disease Questionnaire (PDQ-8)

The PDQ-8 is a disease-specific quality-of-life questionnaire that contains eight items (mobility, activities of daily living, emotional well-being, stigma, social support, cognition, communication, and bodily discomfort), each representing one dimension derived from the PDQ-39 [1]. It was used to measure quality of life [2]. Scores on each item can range from 0 (never or not at all) to 4 (always or cannot do at all). A total score, known as the PDQ-8 Summary Index (PDQ-8 SI, range 0 (normal) to 100 (most severe disability)), was calculated [3]. We interpreted scores in three categories: low (0 – 33), moderate (34 – 67), and high (68 – 100).

Edmonton Symptom Assessment Scale Parkinson Disease (ESAS-PD)

The Edmonton Symptom Assessment Scale Parkinson Disease is a disease specific assessment of late stage PD symptoms. The original ESAS consists of 10 items, whereas the ESAS-PD includes four extra items, namely stiffness, constipation, dysphagia, and confusion [4]. Each item can be scored on a 10-point scale. The maximum score for ESAS-PD is 140. We interpreted scores in three categories: low (0 – 47), moderate (48 – 93), and high (94 – 140).

Functional Assessment of Cancer Therapy – General (FACT-G version 4)

The FACT-G was used to assess the impact of patients' HRQoL, as it provides insight on their physical, social/familial, emotional, and functional well-being. FACT-G was selected because of its ease of administration, relatively short completion time and as it has a high internal consistency [5]. Of, the FACT-G has been widely validated in cancer patients, but not in patients with PD.

The FACT-G total score ranges from 0 to 108, with higher scores signifying a higher HRQoL [5,6]. We interpreted scores in three categories: low (0 - 36), moderate (37 – 72), and high (73 – 108).

2) Questionnaires for family caregivers

Marwit-Meuser Caregiver Grief Inventory Short-Form (MM-CGI-SF)

The MM-CGI-SF is one of the few empirically developed scales that measure pre-death grief [7,8]. Its 18 items are assessed with 5-point Likert scales and summed to generate a total score from 0 – 90. The MM-CGI-SF was originally developed in English. The forward-backward translation technique was used to translate the English version into Dutch. The English items were translated to Dutch by two researchers (HL and MG) independently. They compared their translations and came to a consensus, followed by back-translation by a native speaking independent translator. We interpreted scores in three categories: low (0 – 29), moderate (30 – 59), and high (60 – 90).

Zarit Burden Interview (ZBI)

The Zarit Burden Interview (ZBI) is a 22-item scale that assesses the perceived burden experienced by family caregivers of older patients [9]. ZBI contains five domains: Burden in the Relationship, Emotional Well-being, Social and Family Life, Finances, and Loss of Control over one's life [10]. The items are rated on 5-point Likert scales and summed to generate a total score ranging from 0 to 88, with scores above 60 being considered as high caregiver burden [11]. We interpreted scores in three categories: low (0 – 29), moderate (30 – 59), and high (60 – 88).

Positive Experiences Scale (PES)

The PES is a Dutch instrument that measures positive aspects of family caregiving for patients with a chronic disease [12]. The eight hierarchically-ordered items vary from intrinsic satisfaction and relational enhancement to improvement of competence and social enhancement. Items are scored on a three-point-Likert scale (agree (3), don't agree/don't disagree (2), disagree (1)).

Summed scores range from 8 – 24 [12]. We interpreted scores in three categories: low (0 - 7), moderate (8 – 15), and high (16 – 24).

REFERENCES

[1] Peto V, Jenkinson C, Fitzpatrick R (1998) PDQ-39: a review of the development, validation and application of a Parkinson's disease quality of life questionnaire and its associated measures. *J Neurol* **245**, S10-S14.

[2] Jenkinson C, Fitzpatrick R (2007) Cross-cultural evaluation of the short form 8-item Parkinson's Disease Questionnaire (PDQ-8): results from America, Canada, Japan, Italy and Spain. *Parkinsonism Relat Disord* **13**, 22-28.

[3] Katsarou Z, Bostantjopoulou S, Peto V, Kafantari A, Apostolidou E, Peitsidou E (2004) Assessing quality of life in Parkinson's disease: Can a short-form questionnaire be useful? *Mov Disord* **19**, 308-312.

[4] Miyasaki JM, Long J, Mancini D, Moro E, Fox SH, Lang AE, Marras C, Chen R, Strafella A, Arshinoff R, Ghoche R, Hui J (2012) Palliative care for advanced Parkinson disease: an interdisciplinary clinic and new scale, the ESAS-PD. *Parkinsonism Relat Disord* **18**, S6-9.

[5] Overcash J, Extermann M, Parr J, Perry J, Balducci L (2001) Validity and reliability of the FACT-G scale for use in the older person with cancer. *Am J Clin Oncol* **24**, 591-596.

[6] Cella DF, Tulsky DS, Gray G, Sarafian B, Linn E, Bonomi A, Silberman M, Yellen SB, Winicour P, Brannon J (1993) The Functional Assessment of Cancer Therapy scale: development and validation of the general measure. *J Clin Oncol* **11**, 570-579.

[7] Chan D, Livingston G, Jones L, Sampson EL (2013) Grief reactions in dementia carers: a systematic review. *Int J Geriatr Psychiatry* **28**, 1-17.

[8] Lindauer A, Harvath TA (2014) Pre-death grief in the context of dementia caregiving: a concept analysis. *J Adv Nurs* **70**, 2196-2207.

[9] Zarit SH, Reever KE, Bachpeterson J (1980) Relatives of the impaired elderly - correlates of feelings of burden. *Gerontologist* **20**, 649-655.

[10] Rankin ED, Haut MW, Keefover RW, Franzen MD (1994) The establishment of clinical cutoffs in measuring caregiver burden in dementia. *Gerontologist* **34**, 828-832.

[11] Zarit S, Zarit J (1987) *Instructions for the burden interview*. Pennsylvania State University, University Park.

[12] de Boer AH, Oudijk D, van Groenou MIB, Timmermans JM (2012) Positieve ervaringen door mantelzorg: constructie van een schaal. *Tijd Geront Geriatrie* **43**, 243-254.
